# Supplementary material for: In Vitro Synergy of Colistin in Combination with Meropenem or Tigecycline against Carbapenem-Resistant Acinetobacter baumannii
Source: Antibiotics (Basel). 2021 Jul 20;10(7):880. doi: 10.3390/antibiotics10070880 (PMC8300792; doi:10.3390/antibiotics10070880)
Supplement: Supplementary file 1 [file antibiotics-10-00880-s001.zip › antibiotics-1265440-supplementary.pdf]

antibiotics

MDPI

Supplemental

Article

In vitro Synergy of Colistin in Combination with Meropenem or Tigecycline against Carbapenem-Resistant *Acinetobacter baumannii*

Jacinda C. Abdul-Mutakabbir [1,†], Juwon Yim [1,‡], Logan Nguyen [1], Philip T. Maassen [1], Kyle Stamper [1], Zain Shiekh [1], Razieh Kebriaei [1], Ryan K. Shields [2], Mariana Castanheira [3], Keith S. Kaye [4] and Michael J. Rybak [1,5,6,*]

Table S1. All 50 included isolates plus geographical area.

ARL Isolate #	Source	Geographical Region	Specimen Collection Date	Organism	MEM MIC	COL MIC	TKA Synergy Yes or No?
9008	Blood	DMC, United States	5/22/14	*Acinetobacter baumannii*	64	32	yes
9149	Sputum	DMC, United States	1/8/15	*Acinetobacter baumannii*	64	1	yes
9150	Blood	DMC, United States	2/3/15	*Acinetobacter baumannii*	32	1	yes
9223	Blood	DMC, United States	4/30/15	*Acinetobacter baumannii*	32	0.5	yes
9230	Sputum	DMC, United States	4/28/15	*Acinetobacter baumannii*	32	0.5	yes
9235	Blood	DMC, United States	4/19/15	*Acinetobacter baumannii*	32	1	yes
9314	Endotracheal Aspirate	Beaumont Hospital, United States	8/13/14	*Acinetobacter baumannii*	64	4	no
8407	Sputum	Beaumont Hospital, United States	9/12/14	*Acinetobacter baumannii*	64	4	yes
8410	Tracheal Aspirate	Beaumont Hospital, United Stated	1/31/15	*Acinetobacter baumannii*	32	4	yes
8406	Blood	DMC, United States	6/25/15	*Acinetobacter baumannii*	32	4	yes
9317	Respiratory	DMC, United States	8/7/15	*Acinetobacter baumannii*	32	2	yes
9573	Sputum	DMC, United States	9/25/15	*Acinetobacter baumannii*	64	0.5	yes
10141	Respiratory	DMC, United States	6/7/15	*Acinetobacter baumannii*	32	4	yes
11251	Sputum	DMC, United States	10/18/15	*Acinetobacter baumannii*	32	8	yes
9747	Sputum	Assaf Harofeh Medical Center, Israel	2/1/15	*Acinetobacter baumannii*	8	1	yes

**9748**	**Sputum**	**Assaf Harofeh Medical Center, Israel**	**3/6/15**	***Acinetobacter baumannii***	**8**	**4**	**yes**
**9749**	**Sputum**	**Assaf Harofeh Medical Center, Israel**	**4/1/15**	***Acinetobacter baumannii***	**8**	**8**	**yes**
**9751**	**Sputum**	**Assaf Harofeh Medical Center, Israel**	**5/21/15**	***Acinetobacter baumannii***	**16**	**2**	**no**
**10083**	**Sputum**	**Assaf Harofeh Medical Center, Israel**	**6/3/15**	***Acinetobacter baumannii***	**32**	**2**	**yes**
**9756**	**Sputum**	**Assaf Harofeh Medical Center, Israel**	**6/7/15**	***Acinetobacter baumannii***	**8**	**4**	**yes**
**9760**	**Sputum**	**Assaf Harofeh Medical Center, Israel**	**6/29/15**	***Acinetobacter baumannii***	**8**	**4**	**yes**
**9761**	**Sputum**	**Assaf Harofeh Medical Center, Israel**	**4/27/15**	***Acinetobacter baumannii***	**16**	**1**	**no**
**9767**	**Sputum**	**Assaf Harofeh Medical Center, Israel**	**7/8/15**	***Acinetobacter baumannii***	**8**	**8**	**yes**
**11247**	**Sputum**	**Assaf Harofeh Medical Center, Israel**	**7/11/15**	***Acinetobacter baumannii***	**8**	**8**	**yes**
**9781**	**Sputum**	**Assaf Harofeh Medical Center, Israel**	**8/1/15**	***Acinetobacter baumannii***	**8**	**1**	**yes**
**9784**	**Sputum**	**Assaf Harofeh Medical Center, Israel**	**8/7/15**	***Acinetobacter baumannii***	**8**	**1**	**yes**
**11364**	**Sputum**	**Assaf Harofeh Medical Center, Israel**	**8/23/15**	***Acinetobacter baumannii***	**16**	**8**	**yes**
**9799**	**Sputum**	**Assaf Harofeh Medical Center, Israel**	**8/31/15**	***Acinetobacter baumannii***	**32**	**2**	**yes**
**9802**	**Sputum**	**Assaf Harofeh Medical Center, Israel**	**9/7/15**	***Acinetobacter baumannii***	**32**	**2**	**yes**
**9903**	**Sputum**	**DMC, United States**	**1/24/16**	***Acinetobacter baumannii***	**>64**	**1**	**yes**
**9910**	**Tissue**	**DMC, United States**	**1/15/16**	***Acinetobacter baumannii***	**64**	**2**	**yes**
**9645**	**Sputum**	**Siriraj Hospital, Bangkok, Thailand**	**4/30/16**	***Acinetobacter baumannii***	**>64**	**8**	**yes**
**10409**	**Blood**	**Siriraj Hospital, Bangkok, Thailand**	**5/16/16**	***Acinetobacter baumannii***	**>64**	**4**	**yes**

**10038**	**Sputum**	**Siriraj Hospital, Bangkok, Thailand**	**2/14/16**	***Acinetobacter baumannii***	**>64**	**1**	**yes**
**10042**	**Blood**	**Siriraj Hospital, Bangkok, Thailand**	**2/10/16**	***Acinetobacter baumannii***	**>64**	**2**	**yes**
**9656**	**Blood**	**Siriraj Hospital, Bangkok, Thailand**	**3/16/16**	***Acinetobacter baumannii***	**>64**	**8**	**yes**
**10391**	**Blood**	**Siriraj Hospital, Bangkok, Thailand**	**3/18/16**	***Acinetobacter baumannii***	**>64**	**8**	**yes**
**10054**	**Sputum**	**DMC, United States**	**5/17/16**	***Acinetobacter baumannii***	**>64**	**4**	**yes**
**10404**	**Sputum**	**DMC, United States**	**4/14/16**	***Acinetobacter baumannii***	**>64**	**256**	**yes**
**10062**	**Sputum**	**Siriraj Hospital, Bangkok, Thailand**	**1/21/16**	***Acinetobacter baumannii***	**32**	**16**	**yes**
**10066**	**Blood**	**Siriraj Hospital, Bangkok, Thailand**	**1/30/16**	***Acinetobacter baumannii***	**32**	**4**	**yes**
**10071**	**Blood**	**Siriraj Hospital, Bangkok, Thailand**	**2/5/16**	***Acinetobacter baumannii***	**64**	**16**	**yes**
**10074**	**Blood**	**Siriraj Hospital, Bangkok, Thailand**	**11/14/15**	***Acinetobacter baumannii***	**64**	**16**	**yes**
**10075**	**Sputum**	**Siriraj Hospital, Bangkok, Thailand**	**11/27/15**	***Acinetobacter baumannii***	**64**	**1**	**yes**
**10391**	**Sputum**	**Assaf Harofeh Medical Center, Israel**	**2/14/16**	***Acinetobacter baumannii***	**16**	**32**	**yes**
**10401**	**Sputum**	**Assaf Harofeh Medical Center, Israel**	**2/8/16**	***Acinetobacter baumannii***	**32**	**8**	**yes**
**10910**	**Sputum**	**Siriraj Hospital, Bangkok, Thailand**	**9/18/17**	***Acinetobacter baumannii***	**64**	**8**	**yes**
**10094**	**Sputum**	**Siriraj Hospital, Bangkok, Thailand**	**9/14/15**	***Acinetobacter baumannii***	**>64**	**4**	**yes**
**11367**	**Sputum**	**Assaf Harofeh Medical Center, Israel**	**3/18/18**	***Acinetobacter baumannii***	**16**	**0.5**	**no**
**11542**	**Sputum**	**Siriraj Hospital, Bangkok, Thailand**	**2/4/19**	***Acinetobacter baumannii***	**16**	**0.5**	**no**
